# The effect of hypoxia on facial shape variation and disease phenotypes in chicken embryos

**DOI:** 10.1242/dmm.011064

**Published:** 2013-04-16

**Authors:** Francis Smith, Diane Hu, Nathan M. Young, Alexis J. Lainoff, Heather A. Jamniczky, Emin Maltepe, Benedikt Hallgrimsson, Ralph S. Marcucio

**Affiliations:** 1 Graduate Program in Oral and Craniofacial Sciences, The University of California San Francisco, School of Dentistry, San Francisco, CA 94143, USA; 2 Department of Orthopaedic Surgery, San Francisco General Hospital, The University of California San Francisco, School of Medicine, San Francisco, CA 94110, USA; 3 Department of Cell Biology and Anatomy, The University of Calgary, Faculty of Medicine, Calgary, AB T2N 4N1, Canada; 4 Department of Pediatrics, Program in Biomedical Sciences, The University of California San Francisco, School of Medicine, San Francisco, CA 94110, USA

## Abstract

Craniofacial anomalies can arise from both genetic and environmental factors, including prenatal hypoxia. Recent clinical evidence correlates hypoxia to craniofacial malformations. However, the mechanisms by which hypoxia mediates these defects are not yet understood. We examined the cellular mechanisms underlying malformations induced by hypoxia using a chicken (*Gallus gallus*) embryo model. Eggs were incubated in either hypoxic (7, 9, 11, 13, 15, 17 or 19% O_2_) or normoxic (21% O_2_) conditions. Embryos were photographed for morphological analysis at days 3–6. For analysis of skeletal development, 13-day embryos were cleared and stained with alcian blue and alizarin red for cartilage and bone, respectively. Quantitative analysis of facial shape variation was performed on images of embryos via geometric morphometrics. Early-stage embryos (day 2) were analyzed for apoptosis via whole-mount and section TUNEL staining and immunostaining for cleaved caspase-3, whereas later-stage embryos (days 4–6) were sectioned in paraffin for analysis of cell proliferation (BrdU), apoptosis (TUNEL) and metabolic stress (phospho-AMPK). Results demonstrate that survival is reduced in a dose-dependent manner. Hypoxic embryos displayed a spectrum of craniofacial anomalies, from mild asymmetry and eye defects to more severe frontonasal and cephalic anomalies. Skull bone development was delayed in hypoxic embryos, with some skeletal defects observed. Morphometric analysis showed facial shape variation relative to centroid size and age in hypoxic groups. Hypoxia disrupted cell proliferation and, in early-stage embryos, caused apoptosis of neural crest progenitor cells. Hypoxic embryos also displayed an increased metabolic stress response. These results indicate that hypoxia during early embryonic craniofacial development might induce cellular oxidative stress, leading to apoptosis of the neural crest progenitor cells that are crucial to normal craniofacial morphogenesis.

## INTRODUCTION

Craniofacial malformations, ranging from cleft lip and palate to complex disorders including holoprosencephaly (HPE), affect 84 in 10,000 people worldwide (WHO, 2003). These defects, which can be caused by genetic mutations, environmental factors or combinations of the two, have a mechanistic basis in the alteration of cellular processes during development. For example, mutations in Treacle (TCOF1) induce apoptosis of neural crest progenitor cells, leading to facial hypoplasia (Dixon et al., 2006; Jones et al., 2008), whereas defects in fibroblast growth factor (Fgf) signaling can lead to reduced cell proliferation, causing facial clefting (Abu-Issa et al., 2002; Riley et al., 2007). Teratogenic agents, including cyclopamine, ethanol, nicotine and other drugs, cause similar malformations by altering the survival and proliferation of cells (Webster et al., 2006; Loucks et al., 2007; Perez-Aytes et al., 2008).

A recent clinical report suggests that hypoxia might also be a cause of craniofacial anomalies in humans. Siebert (Siebert, 2007) describes a case of monochorionic, diamniotic twinning in which one twin was normal and the other twin had no heart, which created hypoxia and ischemia due to reversed arterial perfusion. Structural malformations in the acardiac twin included a spectrum of anomalies that resembled HPE (Cohen, 2006): cyclopia, aprosencephaly, cystic hygroma, hypoplastic mandible and small cranium. The investigators suggested that hypoxia and ischemia in the acardiac twin created the HPE phenotype owing to the increased vulnerability of the head to lower levels of oxygen (Siebert, 2007). Thus, hypoxia during pregnancy might affect facial development by increasing apoptosis or reducing cell proliferation. However, the relationship between hypoxia, cellular processes and craniofacial morphogenesis was not tested directly.

The mechanistic link between hypoxia and craniofacial malformations is not well established because the effects of hypoxia on development are poorly understood. In the 1950s and 1960s, a number of researchers (Grabowski and Paar, 1958; Ancel, 1959; Nelsen, 1960; Grabowski, 1961; Grabowski, 1964; Yamamoto and Noguchi, 1964; Grabowski and Schroeder, 1968; Noguchi, 1969; Smith et al., 1969) undertook descriptive studies on the effects of hypoxia on the morphology of chick embryos. Chick embryos were made hypoxic by incubation in an atmospheric mix of air and nitrogen atmosphere, by incubation at high altitude (Smith, 1969), or by shellacking the shells to prevent gaseous diffusion (Ancel, 1959). In all, hypoxia led to high mortality, a variety of craniofacial malformations and, in a small number of embryos, brain defects including exencephaly and anencephaly. However, these studies were qualitative and did not address the cellular mechanisms underlying defects.

**TRANSLATIONAL IMPACT**

**Clinical issue**

Holoprosencephaly (HPE) is a malformation of the developing forebrain and face that, in severe cases, can be associated with miscarriage or stillbirth. Like other craniofacial anomalies, HPE can be caused by genetic mutations, environmental factors or a combination of the two. A recent clinical report implicated hypoxia in the pathogenesis of HPE, and it has been hypothesized that increased apoptosis and reduced cell proliferation due to lower levels of oxygen could underlie the increased vulnerability to malformation. Despite the prevalence of studies on the effects of hypoxia in morphology in avian model systems, the mechanistic link between low-oxygen conditions and craniofacial morphogenesis has not yet been described.

**Results**

In this study, the effects of hypoxia on development were explored in a chicken embryo model. Chicken eggs were incubated in hypoxic conditions and quantitative analysis of facial shape variation was performed. By photographing the embryos between days 3 and 6, the authors tracked the effects on morphogenesis. In addition, developing embryos were analyzed for the presence of apoptosis and changes in cell proliferation. The authors’ analysis indicated that embryos exposed to hypoxic conditions are affected by malformations of the craniofacial complex, including HPE. Hypoxia increased apoptosis, reduced cell proliferation and reduced survival in a dose-dependent manner. Interestingly, the authors observed a spectrum of craniofacial anomalies among embryos that survived chronic hypoxic conditions, suggesting that the phenotypes can be modified by the environment.

**Implications and future directions**

The effects of hypoxia on the early stages of embryonic development have not received widespread attention in recent research into craniofacial morphogenesis. Data from the current study reveals that critical windows of development could be susceptible to reduced oxygen levels, giving rise to disease phenotypes. The results reported here suggest that avoidance of extreme hypoxic conditions, e.g. high altitude or smoking, during pregnancy could protect the developing embryo against craniofacial malformations. However, to fully understand the implications for human development, further translational research using mammalian models is required to elucidate the phenotypic effects of hypoxia on development and to identify the underlying molecular and cellular mechanisms involved.

The goal of this study was to examine the morphological and cellular changes that mediate craniofacial malformations induced by hypoxia in chick embryos. The first question addressed was: does hypoxia alter embryonic craniofacial morphology and, if so, how and to what extent? We examined this by quantifying variation in craniofacial shape and size using two-dimensional geometric morphometrics and comparing the results in hypoxic embryos and normoxic controls. The second question examined was: are abnormal phenotypes caused by changes in cellular behavior? To address this question, cell proliferation and apoptosis were assessed in embryos to determine whether hypoxia reduced cell proliferation and increased cell death. The possibility that hypoxia might cause cellular metabolic stress was also addressed.

## RESULTS

### Hypoxia reduces survival of embryos

**Fig. 1.**
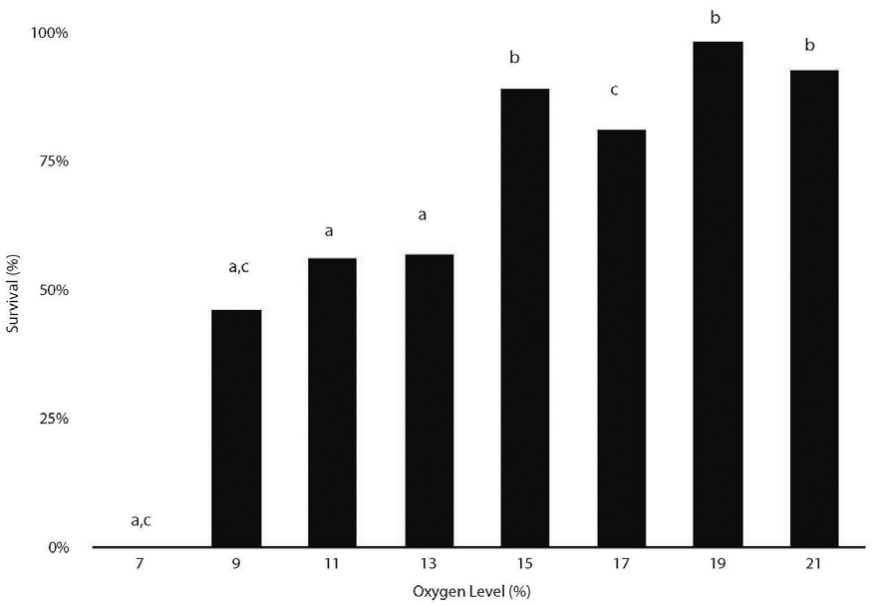
**Hypoxia reduces embryonic survival (at 6 days).** Survival rates for bars marked ‘b’ are significantly higher than those for bars marked ‘a’. Bars marked ‘c’ have survival rates significantly different from each other. *P*<0.05 (Bonferroni adjustment for multiple comparisons). Sample sizes: 7% (*n*=50), 9% (*n*=50), 11% (*n*=50), 13% (*n*=60), 15% (*n*=54), 17% (*n*=42), 19% (*n*=50), 21% (*n*=67).

The survival of embryos exposed to hypoxic conditions for the duration of the experiment (chronic hypoxia: 7, 9, 11, 13, 15, 17 and 19% O_2_) to day 6 was compared to that of normoxic (21% O_2_) controls, in order to assess whether hypoxia increased embryonic mortality. Compared with the 93% survival rate of normoxic embryos, the survival of hypoxic embryos was reduced (Fig. 1) in a stepped pattern. All hypoxic groups were significantly different from the normoxic group (*P*<0.05), and the remaining treatments fell into one of two broad groupings. Embryos in the 15, 19 and 21% O_2_ levels experienced significantly increased survival rates compared with those in the 9, 11 and 13% O_2_ levels (*P*<0.05). In addition, survival at the 17% O_2_ level was significantly increased compared with the 9% O_2_ level (*P*<0.05). There were no significant differences in survival rates within these two broad groupings. There were no survivors at 7% (i.e. the nonviable level) at 6 days. Consequently, there was a plateau below 15% O_2_ at which survival decreased dramatically.

### Hypoxia alters craniofacial morphology

**Fig. 2.**
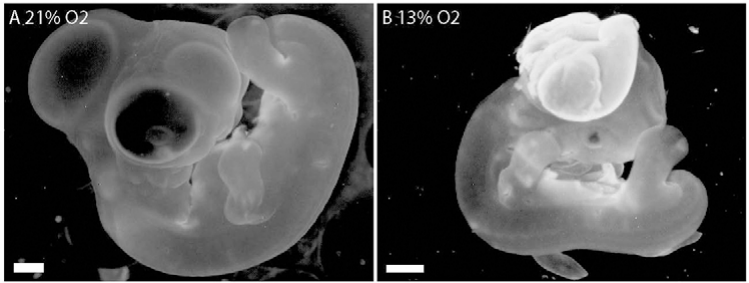
**The head, not the body, is vulnerable to hypoxia-induced malformations.** (A) Normoxic control embryo, day 6. (B) Hypoxic 6-day embryo has a normal body but the head is malformed. Scale bars: 1 mm.

When we examined the morphology of the embryos, we observed that the hypoxic embryos were developmentally delayed and the heads were malformed (supplementary material Table S1), but there were no gross malformations observed in the bodies (Fig. 2). Also, there was no obvious correlation between the level of hypoxia and severity of defects. Compared with normoxic embryos, chronically hypoxic embryos (9, 11 and 13% O_2_) exhibited developmental delay and a spectrum of cephalic and facial anomalies (supplementary material Fig. S1 for 4-day and Fig. S2 for 6-day embryos). Mild defects included asymmetry of the head (supplementary material Fig. S1B,E,F,I,J and Fig. S2B,E,H,I) and eye defects including unilateral or bilateral microphthalmia and anophthalmia (supplementary material Fig. S1B,E,F,I,J and Fig. S2B,E,H,I). More severe malformations included frontonasal and midline defects (supplementary material Fig. S1C,D,G and Fig. S2C,F,J) and brain anomalies including anencephaly, microcephaly and exencephaly (supplementary material Fig. S1C,D,G and Fig. S2C,F,J). A minority of hypoxic embryos were sufficiently malformed to lack any recognizable facial or cephalic structures (supplementary material Fig. S2D,G).

**Fig. 3.**
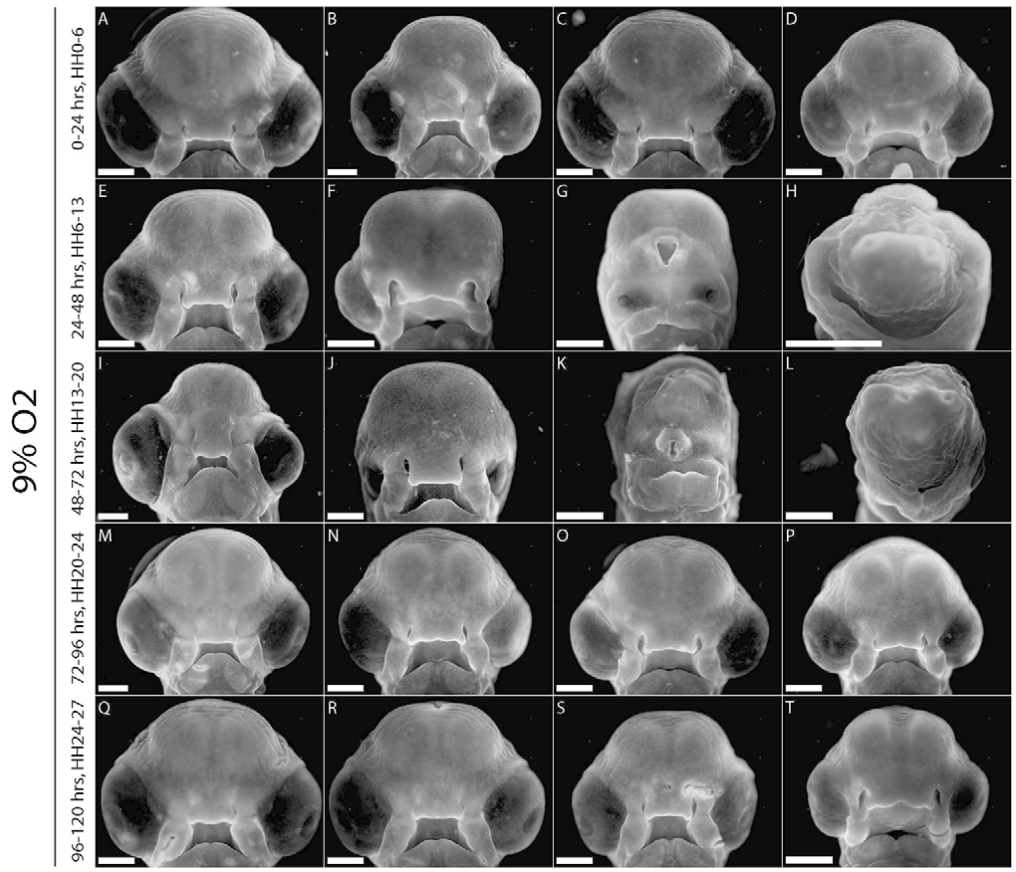
**Acute hypoxia (24-hour windows).** (A–D) Hypoxic from 0–24 hours, no malformations. (E–H) Hypoxic from 24–48 hours, 50% of embryos malformed: (F) left anophthalmia, (G) moderate HPE, (H) no recognizable facial structures. (I–L) Hypoxic from 48–72 hours, 60% of individuals malformed: (I) smaller left eye, (J) bilateral microphthalmia, (K) severe HPE with proboscis, (L) facial obliteration. (M–P) Hypoxic from 72–96 hours; 0% significantly malformed but developmental delay is evident. (Q–T) Hypoxic from 96–120 hours; no malformations but developmental delay apparent. Scale bars: 1 mm.

A similar morphological analysis was performed on acute hypoxic embryos (incubated in 9% O_2_ for 24 hours from 0–24 hours, 24–48 hours, 48–72 hours, 72–96 hours or 96–120 hours of development). All acute hypoxic embryos were developmentally delayed (Fig. 3A–T). None of the embryos in the 0–24 hour hypoxic group was malformed (Fig. 3A–D). 50% of surviving embryos in the 24–48 hour hypoxic group had varying degrees of malformation (Fig. 3F–H), ranging from asymmetry and ophthalmic defects (Fig. 3F), to severe anomalies including HPE (Fig. 3G), and one was sufficiently grossly malformed as to obliterate all facial features (Fig. 3H). In the 48–72 hour group, 60% showed a similar range of malformations (Fig. 3I–L) to those observed in the 24–48 hour group. Embryos in the 72–96 hour (Fig. 3M–P) and 96–120 hour (Fig. 3Q–T) groups showed very mild (Fig. 3N–P,R–T) or no malformations.

**Fig. 4.**
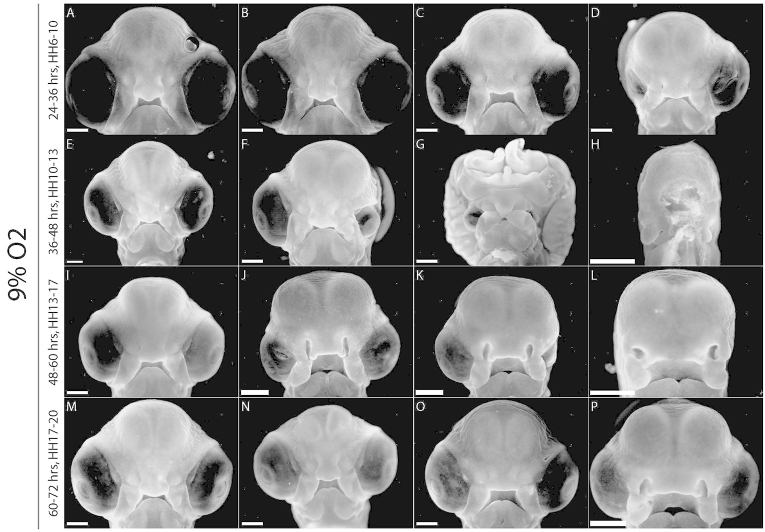
**Acute hypoxia (12-hour windows).** (A–D) Hypoxic from 24–36 hours: (D) right microphthalmia. (E–H) Hypoxic from 36–48 hours, and severe malformations including (F) left microphthalmia, (G) exencephaly, bilateral microphthalmia, hypotelorism and midline defects, and (H) lack of recognizable facial characteristics. (I–L) Hypoxic from 48–60 hours, and milder anomalies including asymmetry and eye defects (J–L). (M–P) Hypoxic from 60–72 hours, and no malformations but developmental delay (O,P). Scale bars: 1 mm.

An additional experiment was performed in which eggs were incubated in 12-hour acute hypoxic periods in 9% O_2_ (24–36 hours, 36–48 hours, 48–60 hours or 60–72 hours). All the hypoxic individuals displayed varying developmental delay (Fig. 4A–P). Only 35% of the first group (hypoxic from 24–36 hours) survived, and these exhibited mild anomalies including unilateral microphthalmia (Fig. 4D). The second group (hypoxic from 36–48 hours) had a higher survival rate (65%), but showed the most severe malformations, including: unilateral microphthalmia (Fig. 4F); exencephaly combined with midline defects, hypotelorism and bilateral microphthalmia (Fig. 4G); and facial obliteration (Fig. 4H). 75% of the third group (48–60 hours) survived, with varying degrees of cephalic asymmetry, microphthalmia and anophthalmia (Fig. 4J–L). The final group, hypoxic from 60–72 hours, had 85% survival and no malformations (Fig. 4M–P).

### Hypoxia alters craniofacial skeletal development

**Fig. 5.**
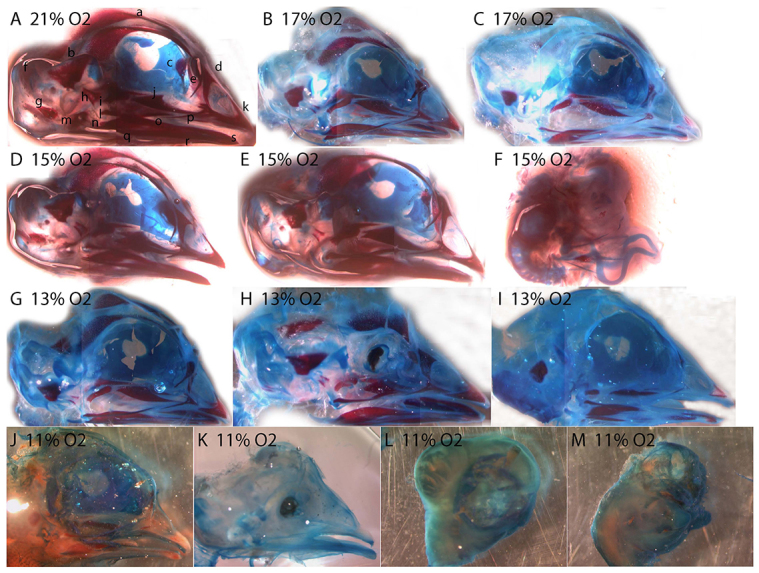
**Hypoxia delays skull development.** (A) Normoxic control, 13 days. (B,C) 17% O_2_. (D–F) 15% O_2_. (G–I) 13% O_2_. (J–M) 11% O_2_. Some individuals (F,H,K) show skeletal anomalies including orbital defects and upper jaw defects. Alizarin red (bone) and alcian blue (cartilage). Bones in panel A: a, frontal; b, parietal; c, ethmoid; d, nasal; e, prefrontal; f, supraoccipital; g, exoccipital; h, quadrate; i, petrosal; j, palatine; k, premaxilla; l, quadratojugal; m, otic; n, articular; o, surangular; p, jugal; q, angular; r, dentary; s, splenial.

Eggs were incubated to day 13 in either normoxia or hypoxia (9, 11, 13, 15 or 17% O_2_), cleared and stained for cartilage and bone. No embryos incubated in 9% O_2_ survived to day 13. Compared with the normal skull structure of normoxic embryos (Fig. 5A), hypoxic embryos showed alterations in ossification to varying degrees in nearly all supramandibular bones (and the frontal, jugal and palatine bones to a lesser degree) (Fig. 5B–M), with a number of embryos displaying orbital and maxillary defects (Fig. 5F,H,K) (supplementary material Table S2). The majority of embryos show delayed ossification (i.e. development appears normal, but ossification is delayed). Severe malformations include gross morphological defects (Fig. 5F).

### Hypoxia leads to abnormal facial shape variation

**Fig. 6.**
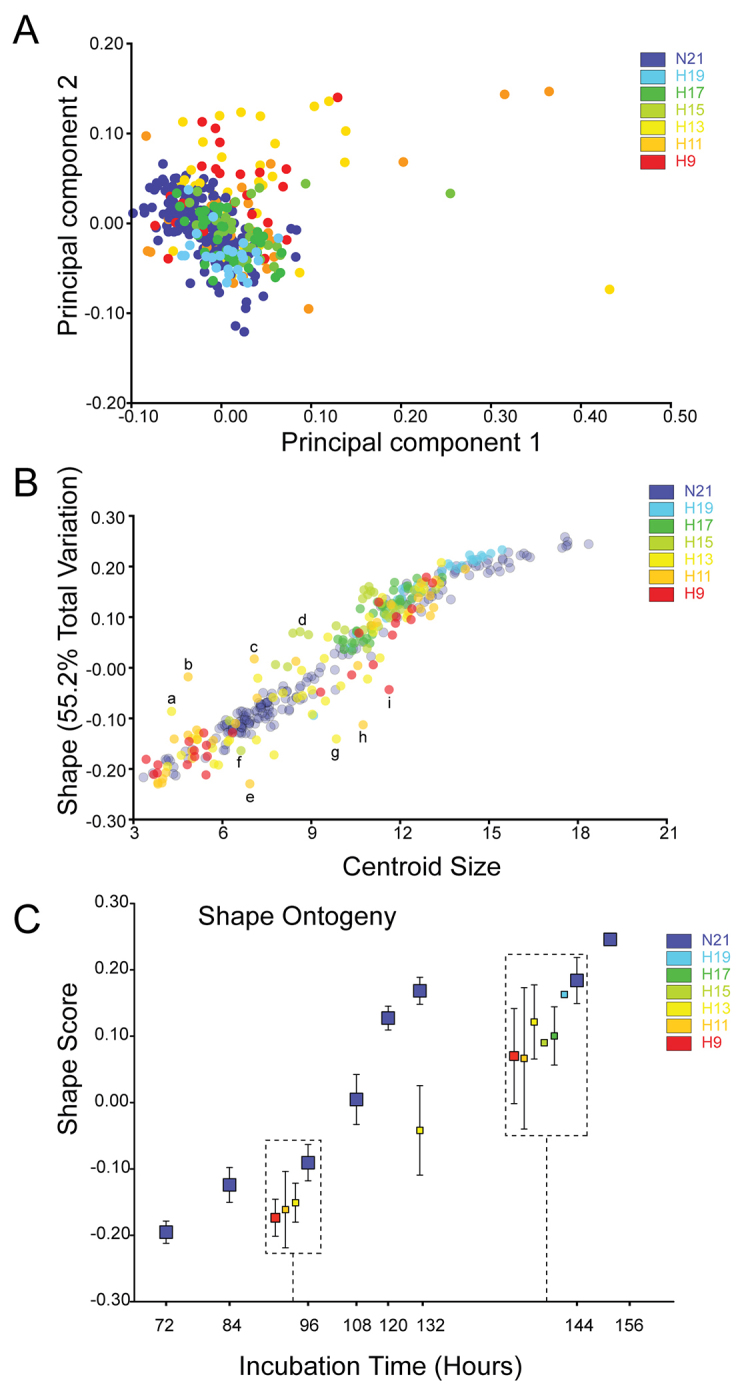
**Abnormal craniofacial shape variation and developmental delay in hypoxic embryos.** (A) Principal components analysis (PCA). Craniofacial shape variation along PC1 and PC2 in normoxic (purple, 21% O_2_) and hypoxic embryos. Normoxic embryos are separated along PC2 (change in size and proportion of craniofacial features), whereas hypoxic embryos are separated along PC1 (abnormal shape variation in forebrain, frontonasal process, nasal pits, eyes and maxillary processes) as well as PC2. (B) Normoxic embryos define a normal growth trajectory, whereas hypoxic embryos deviate from this normal growth curve. (C) Compared with age-matched controls, hypoxic embryos are developmentally delayed. Hypoxic embryos analyzed at each time point are offset in the boxes, and represent the mean ± s.e.m. (See supplementary material Fig. S3 for warped outline diagrams for PC1 and PC2, and supplementary material Fig. S4 for photos of embryos marked a–i in panel B, representing outliers.)

Two-dimensional geometric morphometric analysis was performed on normoxic and hypoxic (9–19% O_2_) embryos to determine whether hypoxia created abnormal facial shape variation. Multivariate regression analysis was performed to quantify the facial shape variation in hypoxic embryos compared with normoxic controls in relation to centroid size and age in hours. Principal components analysis (PCA) of the size residuals demonstrated that, whereas normoxic embryos were separated mainly along principal component 2 (PC2) (size and proportionality of facial features), hypoxic embryos were differentiated along both PC2 and PC1 (shape of eyes, frontonasal process, maxillary processes, nasal pits and forebrain) (Fig. 6A; warped outline diagrams for PC1 and PC2 are shown in supplementary material Fig. S3).

Normoxic embryos were observed to fall along a well-defined growth curve, representing normal facial shape variation in relation to size (Fig. 6B). Hypoxic embryos deviated from this growth trajectory as either inappropriate shape relative to size, or by deviating from the normal curve. Although many fell along the curve, others fell off the curve (examples from Fig. 6B marked a-i shown in supplementary material Fig. S4A–I). There was a roughly dose-dependent effect of hypoxia on developmental delay of the groups of embryos on the curve. The 19% O_2_ embryos did not deviate from the normoxic growth curve in either direction, and were closest to the far right end of the curve. The 17 and 15% O_2_ embryos deviated slightly from the curve, and rested just to the left of the 19% O_2_ group. The 11 and 13% O_2_ groups deviated the most (i.e. comprised the majority of outliers) from the normal trajectory, and were more spread out along the curve. The 9% O_2_ embryos were distributed over most of the length of the curve, but did not show as large a deviation from the normal trajectory as did the 11 and 13% O_2_ embryos (Fig. 6B). In comparison with age-matched normoxic control embryos, hypoxic embryos were developmentally delayed (Fig. 6C). At 96 hours, there was no appreciable difference in delay between the 9, 11 and 13% O_2_ groups of embryos (these were the only hypoxic groups tested at this time point), although all of them were delayed behind the normoxic embryos. However, at 144 hours, the 19% O_2_ embryos were not delayed relative to the normoxic controls. The other groups (9, 11, 13, 15 and 17% O_2_) were delayed compared with the normoxic individuals (Fig. 6C); there was no difference between the hypoxic groups.

### Cell proliferation and apoptosis in hypoxic embryos

**Fig. 7.**
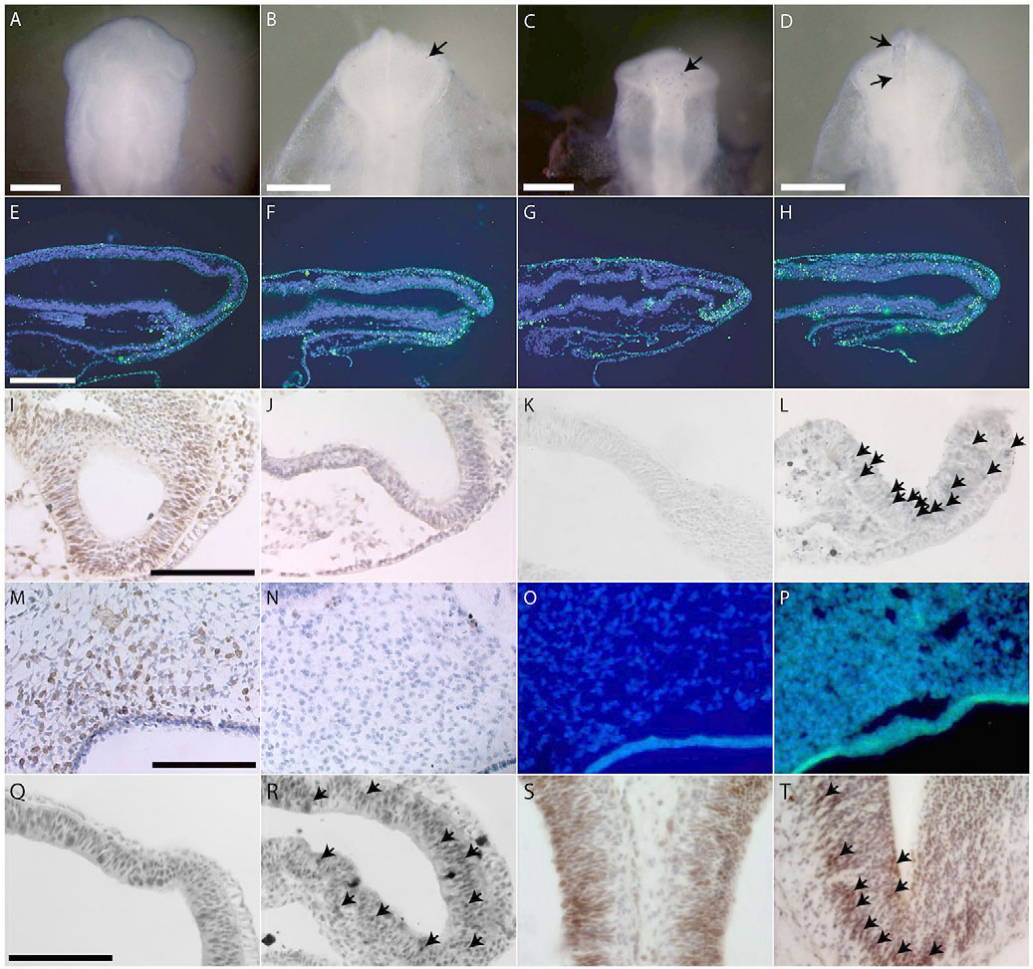
**Cell death, proliferation and metabolic stress in hypoxic embryos.** (A–D) Neural crest progenitor cell death in hypoxic embryos (HH9-10). (A) Normoxic embryo, no cell death. (B,C) Hypoxic embryos with little cell death (arrows). (D) Hypoxic embryo with significant apoptosis in forebrain and neural folds (between arrows). (E–H) Neuroepithelial apoptosis in sections of hypoxic embryos (HH9-10). (E) Normoxic embryo, very little cell death. (F) Hypoxic embryo with some apoptosis in the neuroepithelium in all cephalic regions. (G,H) Hypoxic embryos with significant neuroapoptosis in all cephalic regions. (I-L) Reduced proliferation and increased activity of caspase-3 in neuroepithelial cells in hypoxic embryos (HH9-10). Healthy proliferation of neuroepithelial cells (BrdU-positive cells) is evident in the normoxic embryo (I), whereas there is reduced proliferation in the hypoxic neuroepithelium (J). Compared with the normoxic control (K), there are caspase-3-positive cells (arrows) present in the neuroepithelium of the hypoxic embryo (L). (M–P) Cell proliferation and apoptosis at later stages (6 days). (M) Large region of BrdU-positive cells in the frontonasal mesenchyme of a normoxic control embryo. (N) Lack of BrdU-positive cells in mesenchyme of a hypoxic embryo. (O,P) No apoptosis in the frontonasal mesenchyme of normoxic (O) or hypoxic (P) embryos. (Q–T) Metabolic stress in hypoxic embryos. (Q,R) 2 days, (S,T) 6 days. Whereas pAMPK staining is widespread in both normoxic and hypoxic embryos (Q–T), there is intensified staining (arrows) in the neuroepithelium of (R) hypoxic 2-day and (T) 6-day embryos. Scale bars: 250 μm (A–E,M); 1 mm (I,Q).

Cell behavior analyses were undertaken to explain whether the abnormal phenotypes observed in hypoxic embryos could be explained by changes in cell proliferation and death in the cephalic and facial tissues. We analyzed cell death in embryos incubated in 9 and 13% oxygen, because 9% was the lowest dose of oxygen used and 13% was the threshold for malformations. Compared with normoxic embryos (Fig. 7A), which showed no apparent apoptosis, hypoxic embryos (at 13% O_2_) exhibited varying amounts of neural crest progenitor cell death in the dorsal neural tube (Fig. 7B–D). Whereas some showed little apoptosis (Fig. 7B,C), others displayed larger amounts of cell death in the forebrain and neural folds (Fig. 7D). To confirm whether the apoptosis seen in whole-mount TUNEL staining was indeed neuroepithelial in origin, fluorescent TUNEL staining was undertaken in sections of normoxic and hypoxic (13% O_2_) embryos from the same age. In comparison with the small amount of staining in control embryonic neuroepithelium (Fig. 7E), hypoxic embryos displayed variation in the amount of neuroepithelial cell death. Whereas some hypoxic embryos showed some scattered staining in the neuroepithelium, ectoderm and mesoderm (Fig. 7F), others showed more frequent, widespread apoptosis in all cephalic regions of the neuroepithelium, as well as in the mesoderm and ectoderm (Fig. 7G,H). At this same time point, hypoxic embryos exhibited reduced cell proliferation in the neuroepithelial tissues of the head (Fig. 7J), compared with normoxic controls (Fig. 7I). In comparison with normoxic embryos (Fig. 7K), hypoxic embryos (9% O_2_) also showed more cleaved-caspase-3-positive cells, indicative of increased cell death (Fig. 7L). In a later stage of development (6 days), bromodeoxyuridine (BrdU) incorporation in adjacent sections of the same embryos was used to assess cell proliferation in frontonasal mesenchyme. Compared with normoxic 6-day embryos (Fig. 7M), age-matched hypoxic embryos showed reduced cell proliferation (Fig. 7N). Neither normoxic nor hypoxic embryos showed any evidence of apoptosis in the frontonasal mesenchyme (Fig. 7O,P) via TUNEL staining.

### Metabolic stress in hypoxic embryos

Phenotypic variability in the sample suggested a generalized effect of hypoxia on craniofacial morphology; thus, the possibility of a cellular metabolic stress response to hypoxia was addressed. One marker of metabolic stress is the phosphorylated (activated) form of AMP-activated protein kinase (AMPK), a regulator of the ATP:AMP energy balance in the cell, activated by stressors including hypoxia. Although pAMPK staining was widespread in both normoxic and hypoxic embryos (Fig. 7Q–T), we observed more cells with greater intensity of staining in the neuroepithelial tissues of hypoxic embryos (Fig. 7R,T) compared with normoxic controls (Fig. 7Q,S) at both 2 and 6 days.

## DISCUSSION

Craniofacial malformations are disfiguring conditions that can be caused by genetic mutations, environmental factors or a combination of both. One particular environmental factor that is implicated in craniofacial anomalies is hypoxia during facial morphogenesis. Despite clinical and experimental evidence of a correlation between embryonic hypoxia and craniofacial defects, the mechanisms by which hypoxia mediates craniofacial malformations are not yet understood. The goal of this work was to quantify hypoxia-induced morphological and cellular changes in a chick model, with the hypothesis that malformations are generated by changes in normal cellular processes. We found that chronic hypoxia led to a reduced survival rate, developmental delays, and a spectrum of cephalic and facial anomalies. Hypoxia also caused neural crest progenitor apoptosis in early-stage embryos and reduced frontonasal mesenchymal cell proliferation at later stages, suggesting a causal link between hypoxia, rates of growth and morphological outcomes.

### Hypoxia causes a range of malformations

There was a wide spectrum of cephalic and facial anomalies among those embryos that survived chronic hypoxic conditions. The milder, and more common, malformations consisted of cephalic asymmetry and eye defects including unilateral or bilateral anophthalmia and microphthalmia. A small group of embryos had frontonasal anomalies including an absent midline. The most severe defects, present in a small number of embryos, were neural tube defects including anencephaly, microcephaly and exencephaly. These disorders in some cases were combined with midline defects (including absent frontonasal process). The rarest malformations were so severe that no facial features were recognizable. This finding raised the possibility that the embryos that died were too severely malformed for viability in hypoxia. Hypoxia most likely exerts a selective pressure against survival of the most severely defective embryos. Among the hypoxic embryos, no correlation between severity of malformations and level of oxygen was observed. The wide variability of phenotypes indicated incomplete penetrance.

The experiments with acute hypoxia helped us identify critical periods of development that were susceptible to hypoxic insult. Up to 24 hours of development, survivability was severely compromised, but after this time hypoxia affected growth of embryos and morphogenesis of the face. Between 24 and 48 hours of development, hypoxia produced the most severe anomalies, including rare cases of HPE with a proboscis and even rarer instances of total obliteration of facial characteristics. Further experiments with acute hypoxia for 12 hours narrowed down the critical vulnerable window for hypoxia to between 24 and 36 hours of development, with only 35% of hypoxic individuals surviving. The window of hypoxia between 36 and 48 hours produced embryos with the most severe malformations, including neural tube defects. Later 12-hour windows of acute hypoxia produced improved levels of survival and reduced severity of malformations. Interestingly, this critical hypoxic window occurs during the period in which the neural crest cells are generated and begin to delaminate and migrate, suggesting that these cells are particularly vulnerable to hypoxic stress at this time point. Many craniofacial malformations and syndromes are neurocristopathies resulting from disorders of neural crest development, proliferation and migration, and the malformations observed here resemble these phenotypes.

From Siebert’s findings (Siebert, 2007) of HPE-like facial and cephalic defects in an acardiac human twin fetus, it was hypothesized that hypoxia led to HPE. Here, we observed a range of malformations that included HPE among a range of other malformations. Although the phenotypes were too varied to identify the molecular and cellular causes of each phenotype, the cell death that is distributed throughout regions of the neuroepithelium around the time of neural crest generation might explain a majority of the malformations observed here. For example, increased apoptosis that affects the midline of the ventral forebrain might lead to HPE-like phenotypes in some embryos (Fig. 7G,H), whereas in other embryos decreased survival of neural crest progenitor cells might lead to hypoplasia of the facial primordia (Fig. 7C,D). These ideas are supported by our observations that there is a critical window wherein hypoxia exerts teratogenic effects on the face. This window occurs between 24 and 48 hours of development. Hypoxia during this period produced the most severe anomalies, including rare cases of HPE with a proboscis and even rarer instances of total obliteration of facial characteristics. Further experiments with acute hypoxia for 12 hours narrowed down the critical vulnerable window for hypoxia to between 24 and 36 hours of development, with only 35% of hypoxic individuals surviving. The window of hypoxia between 36 and 48 hours produced embryos with the most severe malformations including neural tube defects. This period of development corresponds to the timing of neural crest generation and initial forebrain patterning. Later 12-hour windows of acute hypoxia produced improved levels of survival and reduced severity of malformations. Combined, these results suggest that the extent and distribution of neuroepithelial cell death could contribute to a wide range of structural disease phenotypes in the skull.

A possible cellular level model for the effect of hypoxia on the craniofacial and brain development in embryos can be described as follows. Under hypoxic conditions, particularly during the critical stage of development of the neural crest and its delamination, the reduction of oxygen available to these cells triggers a metabolic stress response. A functional electron transport chain is required to sense oxygen levels and trigger apoptosis in response to hypoxic conditions (Jacobson et al., 1993). Evidence that hypoxia-induced cell death was regulated by the intrinsic (mitochondrial) pathway of apoptosis (reviewed in Shroff et al., 2007) began to emerge with studies demonstrating that anti-apoptotic Bcl-2 and Bcl-xL proteins could protect cells exposed to severe hypoxia (Shimizu et al., 1995). Additionally, cells lacking other Bcl-2 family members such as Bax and Bak are also protected from anoxia-induced cell death (McClintock et al., 2002). During oxygen deprivation, Bax translocates from the cytoplasm to mitochondria where it triggers cytochrome *c* release and subsequent cell death (Saikumar et al., 1998). Apoptosis induction is not prevented in the absence of Bid activity, however, indicating that the extrinsic pathway of apoptosis is dispensable for severe hypoxia-induced cell death (Brunelle et al., 2007). This results in a neurocristopathy, the basis for the neural tube defects and other craniofacial malformations to varying degrees among hypoxic embryos.

Interestingly, in humans, hypoxia associated with smoking results in a significant increase in craniofacial anomalies (Savitz et al., 1991) and our results indicate that these structures are also particularly sensitive to oxygen availability during chick embryonic development. This suggests the possibility that neural crest cells have higher metabolic demands, and thus might be particularly sensitive to changes in mitochondrial electron transport chain function. In mice as well as birds, chemical inhibition of electron transport chain function produces a high incidence of cleft lip and palate (Trasler et al., 1978; Landauer and Sopher, 1970). Additionally, maternal hypoxia in susceptible mouse strains (Bronsky et al., 1986; Millicovsky and Johnston, 1981) promotes cleft lip and palate formation. By the time of primary palate formation, roughly half of all ATP is generated by mitochondrial oxidative phosphorylation (Hunter and Sadler, 1988), rendering the tissues that form it sensitive to oxygen deprivation.

### Could hypoxia or metabolic stress potentiate neurocristopathies?

More work in mammalian models is necessary for a full understanding of the potential teratogenic effects of low oxygen levels during critical stages of embryogenesis. One important difference between chick and mammalian embryos is the altered oxygen environment *in ovo* versus *in utero*. Whereas chick embryos develop in atmospheric oxygen (21%), mammalian development occurs in a physiological hypoxia (1–5% O_2_). Hypoxia is therefore a relative term among different organisms, and even cells within an organism experience and respond to oxygen levels in unique ways. Thus, similar malformations are observed in mammalian embryos that develop in hypoxic environments, but the threshold of hypoxia for these embryos is lower. Furthermore, oxygenation of mammalian embryos relies on a gas exchange interface provided by the placenta (Maltepe et al., 2010). Although the placenta can respond to changes in environmental oxygen tensions by increasing the surface area for exchange, clinical conditions such as preeclampsia or cigarette smoking impair placental gas exchange resulting in significant fetal hypoxia (Burton, 2009). Importantly, deviations below ‘normal’ oxygen tensions in either setting are detrimental to development in both birds and mammals, validating the use of the chick model to understand the effects of oxygen tension on vertebrate development.

### Clinical implications and future directions

Recent clinical evidence, as well as previous experimental studies, have correlated embryonic hypoxia with craniofacial and brain malformations. However, this correlation has not been further explored to date, nor have the mechanisms underlying hypoxia-induced facial and cephalic defects become understood. Therefore, the goal of this study was to examine the morphological changes and possible cellular mechanisms responsible for craniofacial anomalies caused by hypoxia. Although this work was limited to the study of model organisms, our results indicate that avoiding pathological hypoxia, either due to altitude or environmental factors (e.g. smoking), particularly during critical early windows of embryonic and fetal development, might help prevent craniofacial malformations. It also suggests that intrinsic defects to oxygen metabolism or extrinsic limits to oxygen availability might be a common unifying factor that contributes to a range of craniofacial defects. As the mechanisms behind hypoxia-induced malformations are better understood, possibilities exist for potential cellular-based and molecular or anti-oxidant therapies that can be employed in at-risk populations during early embryonic development before malformations are generated.

## MATERIALS AND METHODS

### Chick embryos

#### Chronic hypoxia

Fertile White Leghorn chicken eggs (Petaluma Farms, Petaluma, CA) were incubated in a humidified chamber (Hova-Bator, GQF Manufacturing, Savannah, GA) at 37.5°C for the duration of the experiment. The day on which the eggs were placed in the incubator was designated as day 0. Nitrogen levels were increased to generate hypoxic conditions. The level of oxygen in the chamber was titrated to 7, 9, 11, 13, 15, 17 or 19% using an oxygen sensor/controller (Proox, Biospherix, Lacona, NY). As a control, another group of eggs was incubated in normoxic (21% O_2_) conditions.

#### Acute hypoxia

To narrow down the putative critical time window of vulnerability to hypoxia, groups of eggs were incubated initially in normoxia, then transferred to 9% oxygen for 24 hours on day 0 (0–24 hours), 1 (24–48 hours), 2 (48–72 hours), 3 (72–96 hours) or 4 (96–120 hours), and returned to normoxia until collection on day 6. In a subsequent experiment, groups of eggs were incubated in postnormoxic acute hypoxic periods of 12 hours (24–36 hours, 36–48 hours, 48–60 hours or 60–72 hours of development), then returned to normoxia and collected on day 6.

Chick embryos were collected on days 2 through 6 and staged according to the Hamburger and Hamilton (HH) staging system (Hamburger and Hamilton, 1951). For cartilage and bone analyses, a number of embryos were incubated to day 13 in normoxic, 9%, 11%, 13%, 15% and 17% O_2_ conditions. Embryos were removed from extraembryonic membranes, sacrificed by decapitation, washed in 1× PBS (phosphate buffered saline), and fixed overnight in 4% paraformaldehyde in 1× PBS at 4°C.

### Statistical analysis of embryonic survival

A pairwise Fisher’s Exact Test was used to determine the significance of differences in the number of embryos that survived at each oxygen level (Fig. 1). A post-hoc Bonferroni correction was performed on the calculated *P*-values to account for multiple comparisons.

### Gross morphological analyses

Immediately following collection and fixation, embryos were washed in 1× PBS and stained for fluorescent microphotography in 0.01% ethidium bromide in 1× PBS. Embryos were next placed in a Petri dish with the face anterior and the middle of the eyes even with the nasal pits. Each was photographed using a Texas Red fluorescent filter on a Leica MZFLIII dissecting microscope with a Leica LEI-750 camera (Leica Microsystems, Germany). The photographs were acquired using Adobe Photoshop imaging software (Adobe, San Jose, CA).

### Analysis of cartilage and bone development

Embryos (normoxic control, 9%, 11%, 13%, 15% and 17% O_2_) were collected on day 13, sacrificed by decapitation, and the heads washed in 1× PBS. Heads were fixed overnight in 4% PFA at 4°C. After washing in 1× PBS, the heads were stripped of eyes and skin. Embryos were dehydrated in a graded ethanol series and stained for cartilage with alcian blue in 1% glacial acetic acid in ethanol. After rehydration, specimens were trypsinized to clear the soft tissues of alcian blue stain. Embryos were next stained for bone with alizarin red in 0.5% potassium hydroxide in water and cleared in an ascending graded glycerol series to 100% glycerol. These were visualized and photographed using a Leica MZFLIII dissecting microscope with a Leica LEI-750 camera (Leica Microsystems, Germany) and digitized using Adobe Photoshop imaging software (Adobe, San Jose, CA).

### Geometric morphometrics

Facial landmarks, as previously designated by Chong and colleagues (Chong et al., 2012), were located on digital photographs in NIH ImageJ (NIH, Bethesda, MD). These two-dimensional (*x*,*y*) coordinate data were analyzed in MorphoJ (Klingenberg, 2011). After, Procrustes superimposition was used to analyze for facial shape variation between individuals in normoxic and hypoxic groups. Multivariate linear regression was performed to quantify facial shape variation due to centroid size (mean size of each embryo about a central point determined by Procrustes superimposition), age (hours) and stage (HH), and also to remove the effect of size heterogeneity on shape by use of the residuals. Size-corrected PCA was used to analyze for facial shape variation between individuals in normoxic and hypoxic groups.

### Assessment of cell proliferation

Embryos were injected intravascularly 20 minutes prior to harvest with 1 μl BrdU labeling reagent (Zymed, South San Francisco, CA). These were subsequently fixed, dehydrated, embedded and sectioned in paraffin. BrdU incorporation was assessed by immunohistochemistry and detected with diaminobenzidine (DAB) followed by counterstaining all nuclei with hematoxylin according to the kit manufacturer’s instructions (Invitrogen, Camarillo, CA). Sections were visualized and photographed with a Leica DM5000B microscope and LEI-750 camera (Leica Microsystems, Germany).

### Analysis of apoptosis

To assess cell death in whole-mounts, embryos were fixed, dehydrated in a graded ethanol series, rehydrated and treated with proteinase K digestion. Subsequently, they were post-fixed in 4% PFA + 0.1% glutaraldehyde. The embryos were stained in TDT buffer, TDT enzyme and AP converter from the Roche *In Situ* Cell Death Detection Kit, AP (Roche Applied Science, Indianapolis, IN). To enable visualization of dead cells in whole-mount embryos, they were stained with NBT and BCIP. Whole-mount-stained embryos were photographed on a Leica MZFLIII dissecting microscope with a Leica LEI-750 camera (Leica Microsystems, Germany).

For analysis of cell death in sections, embryos were sectioned in paraffin and TUNEL staining was performed on sections according to the TUNEL kit manufacturer’s protocol (Roche Applied Science, Indianapolis, IN) to examine nuclear DNA fragmentation. Stained sections were imaged by fluorescent microscopy using a Leica DM5000B microscope and LEI-750 camera (Leica Microsystems, Germany). All nuclei were stained (blue) using Hoechst dye to enable visualization using the A cube. The L5 cube was used to visualize dead cells (green). The TUNEL staining on additional sections was continued with AP converter (Roche Applied Science, Indianapolis, IN) and BM purple AP substrate (Roche Applied Science, Indianapolis, IN). Those sections stained via AP conjugation were visualized by brightfield microscopy.

Further analysis for apoptosis was undertaken through immunohistochemical staining for cleaved caspase-3, an important part of the apoptotic pathway. Paraffin sections of 2-day normoxic and hypoxic embryos were stained with a rabbit monoclonal primary antibody against cleaved caspase-3 (1:100; Cell Signaling Technology, Beverly, MA) and a goat anti-rabbit secondary IgG (H^+^L) antibody (1:100). Staining was detected by diaminobenzidine. Stained sections were visualized by brightfield microscopy.

### Metabolic analysis

Analysis for cellular metabolic stress in response to hypoxia was performed via immunohistochemistry for AMP-activated protein kinase, an essential regulator of the AMP-ATP balance. Paraffin sections were stained with a rabbit monoclonal antibody against phospho-AMPK (Thr172) (1:100; Cell Signaling Technology, Beverly, MA) and a goat anti-rabbit secondary IgG (H^+^L) antibody (1:100). Staining was detected with diaminobenzidine. Visualization was performed via brightfield microscopy.

## Supplementary Material

Supplementary Material
