# Supplementary material for: The effect of hypoxia on facial shape variation and disease phenotypes in chicken embryos
Source: Dis Model Mech. 2013 Apr 16;6(4):915–24. doi: 10.1242/dmm.011064 (PMC3701211; doi:10.1242/dmm.011064)
Supplement: Supplementary Material [file supp_6_4_915__index.html]

The effect of hypoxia on facial shape variation and disease phenotypes in chicken embryos — The effect of hypoxia on facial shape variation and disease phenotypes in chicken embryos — Supplementary Material 

# The effect of hypoxia on facial shape variation and disease phenotypes in chicken embryos

## DMM011064 Supplementary Material

**Files in this Data Supplement:**

- **Supplementary Material PDF**
