# Supplementary material for: The effect of hypoxia on facial shape variation and disease phenotypes in chicken embryos
Source: Dis Model Mech. 2013 Apr 16;6(4):915–24. doi: 10.1242/dmm.011064 (PMC3701211; doi:10.1242/dmm.011064)
Supplement: Supplementary Material [file supp_011064_DMM011064.pdf]

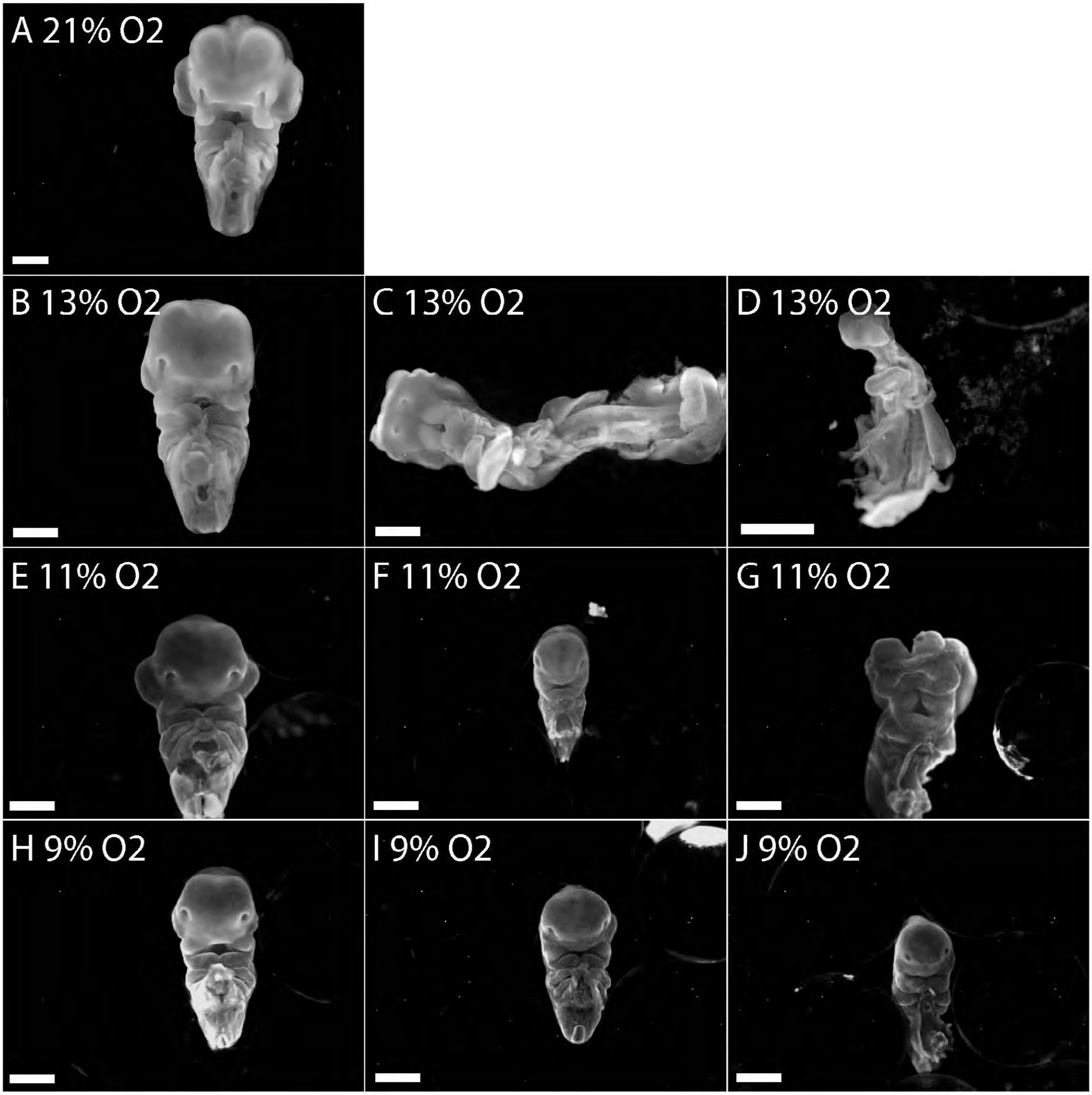

Supplemental Fig. 1. Hypoxia creates abnormal craniofacial morphology (day 4). (A) Normoxic control, day 4, (B-D) 13% O_2, (E-G) 11% O2, (H-J) 9% O_2. (B,E,F,H,I,J) Developmental delay, asymmetry, and eye defects. (C,D,G) Severe anomalies include neural tube defects and midline defects. Scale bar: 1mm.

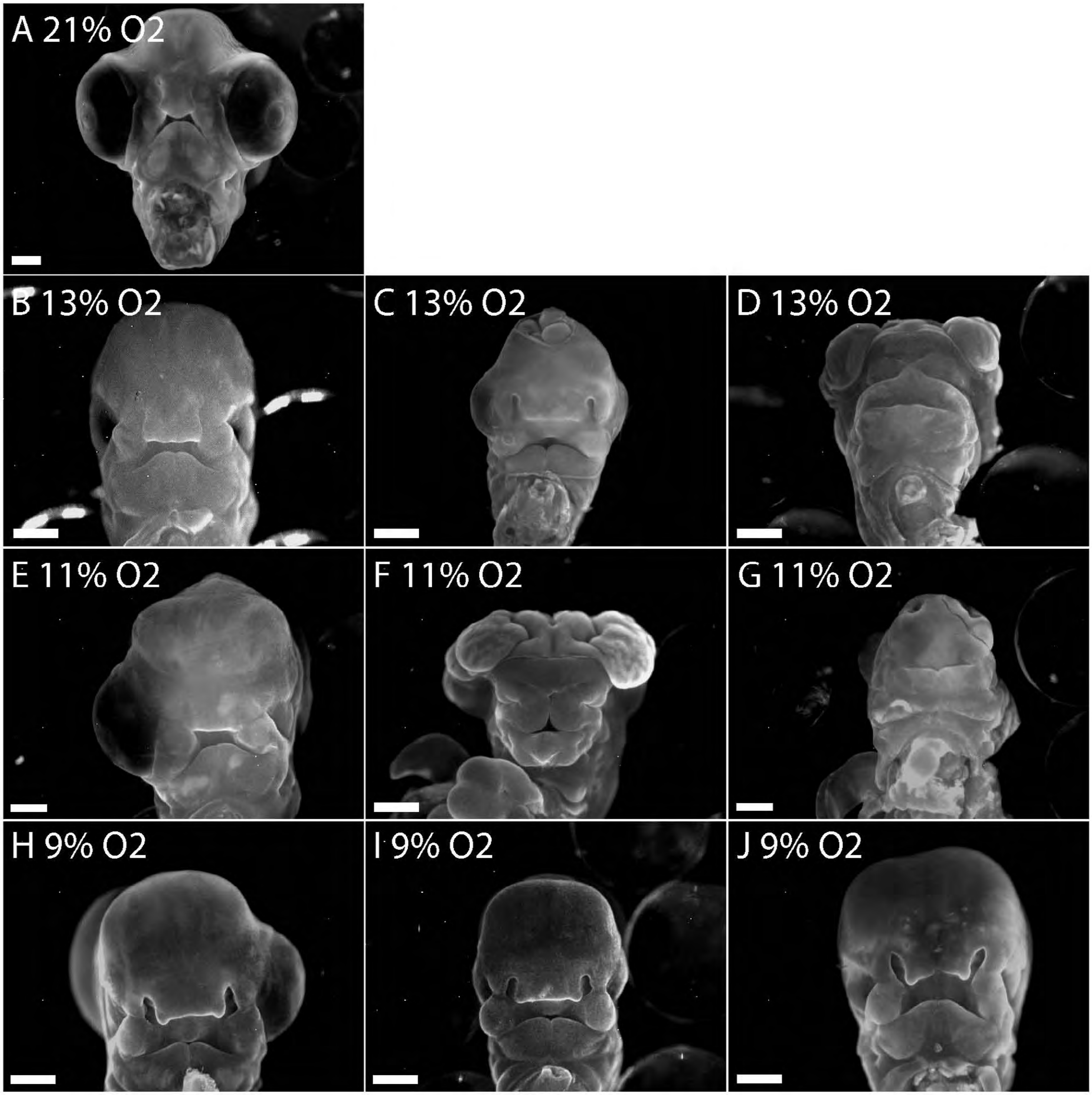

Supplemental Fig. 2. Hypoxia creates abnormal craniofacial morphology (day 6). (A) Normoxic control, day 6, (B-D) 13% O2, (E-G) 11% O2, (H-J) 9% O2. (B,E,H,I) Developmental delay, asymmetry, and eye anomalies. (C,F,J) Severe malformations include brain and neural tube defects and midline anomalies. Two embryos (D,G) are sufficiently malformed to lack recognizable facial landmarks. Scale bar: 1mm.

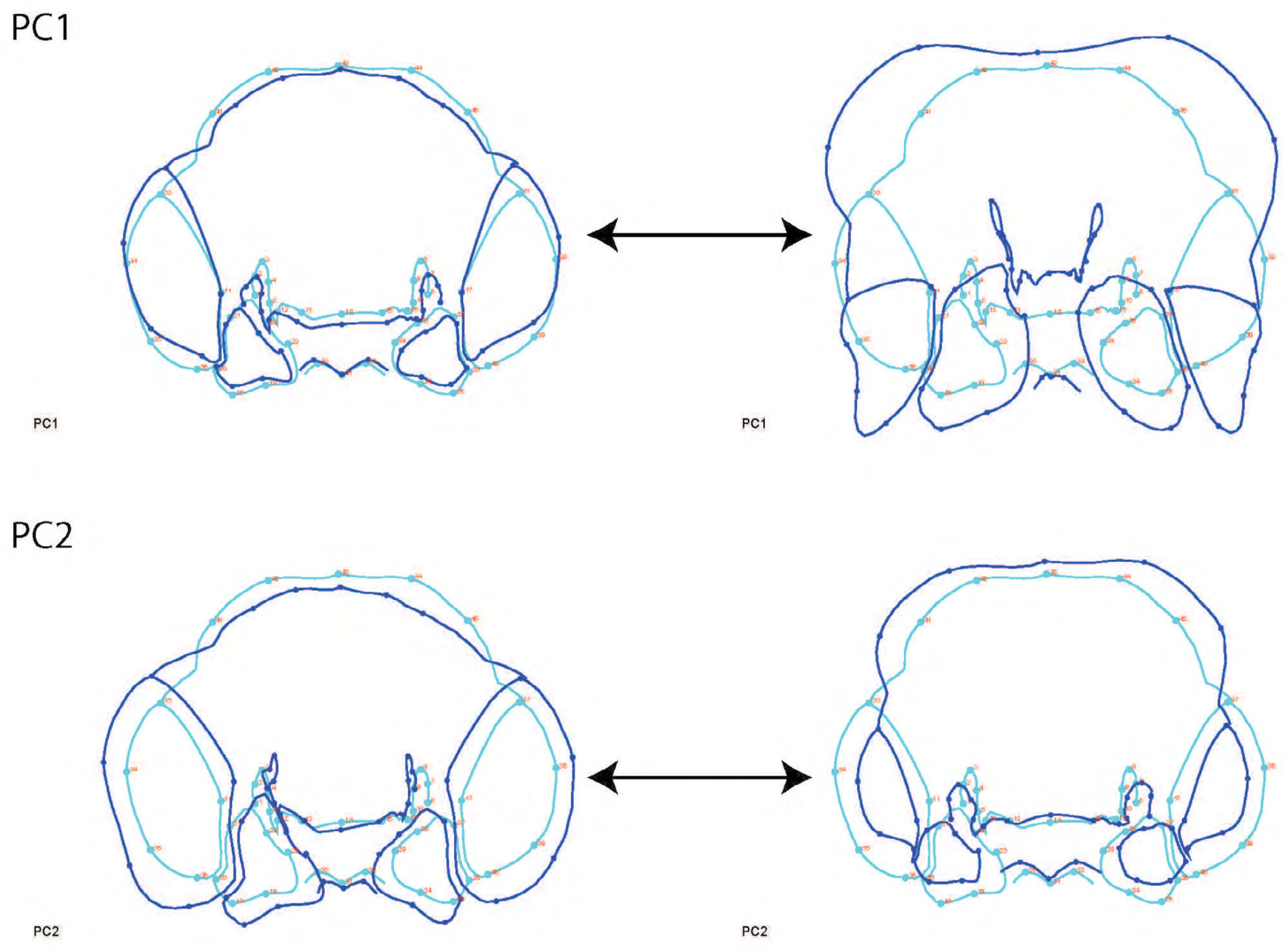

Supplemental Fig. 3. Examples of outliers in centroid size regression, Fig. 6B.

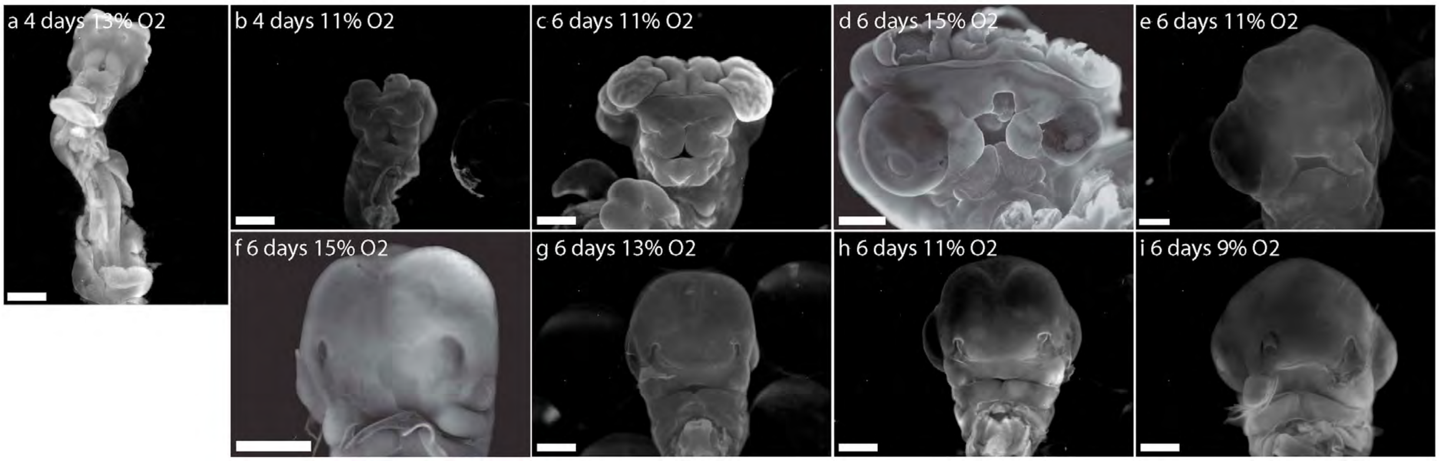

Supplemental Fig. 4. Warped outline diagrams for principal components PC1 and PC2, Fig. 6A. PC1 represents abnormal shape variation in forebrain, frontonasal process, maxillary processes, nasal pits, and eyes. PC2 represents change in size and proportionality of craniofacial features. Light blue wires represent the mean shape of all embryos and dark blue wires represent the extreme shape along each axis of the PCs.

Oxygen Level (%O2)	No developmental delay	Developmental delay only	Unilateral eye defect only	Bilateral eye defect only	Exencephaly	Severe dysmorphology	HPE	Total survivors
**9% O2 (4 days)**	0	14	5	1	0	3	0	23
**9% O2 (6 days)**	0	12	6	2	0	4	0	23
**11% O2 (4 days)**	0	9	3	1	1	1	0	15
**11% O2 (6 days)**	0	20	1	0	3	4	0	28
**13% O2 (4 days)**	0	10	6	2	0	3	0	21
**13% O2 (6 days)**	0	27	2	4	1	0	0	34
**15% O2 (6 days)**	0	43	3	0	0	2	0	48
**17% O2 (6 days)**	0	33	1	0	0	0	0	34
**19% O2 (6 days)**	36	12	0	0	0	1	0	49

Supplemental Table 1: Prevalence of phenotypes in surviving hypoxic embryos. While the majority of phenotypes are developmentally delayed, a number of embryos in each hypoxic group display a range of defects from simple eye defects to gross dysmorphology. Unilateral eye defects are the second most prevalent anomalies. Severe phenotypes are more prevalent in groups of embryos incubated in lower oxygen levels. Abreviation: HPE=holoprosencephaly.

Oxygen level (%O2)	Delayed ossification	Mild malformation	Severe malformation	Total sample
13% O2	18	1	1	20
11% O2	4	1	7	12
15% O2	5	1	2	8
17% O2	9	0	0	9

Supplemental Table 2: Prevalence of altered ossification in surviving hypoxic embryos. The majority of embryos show delayed ossification, where bone development appears normal but appropriate morphology appears later in development. Mild malformations include some bone loss and mild defects of cartilaginous elements. Severe malformations include total absence of bone and gross morphological defects.
